# Acquisition of extended spectrum beta-lactamase-producing enterobacteriaceae in neonates: A community based cohort in Madagascar

**DOI:** 10.1371/journal.pone.0193325

**Published:** 2018-03-01

**Authors:** Perlinot Herindrainy, Mamitiana Alain Noah Rabenandrasana, Zafitsara Zo Andrianirina, Feno Manitra Jacob Rakotoarimanana, Michael Padget, Agathe de Lauzanne, Awa Ndir, Elsa Kermorvant-Duchemin, Benoit Garin, Patrice Piola, Jean-Marc Collard, Didier Guillemot, Bich-Tram Huynh, Elisabeth Delarocque-Astagneau, for the BIRDY study group

**Affiliations:** 1 Epidemiology Unit, Institut Pasteur de Madagascar, Antananarivo, Madagascar; 2 Experimental Bacteriology Unit, Institut Pasteur de Madagascar, Antananarivo, Madagascar; 3 Peadiatric Ward, Centre Hospitalier de Soavinandriana, Antananarivo, Madagascar; 4 Biostatistics, Biomathematics, Pharmacoepidemiology and Infectious Diseases Unit, Institut Pasteur, INSERM, UVSQ, Paris, France; 5 Epidemiology and Public Health Unit, Institut Pasteur du Cambodge, Phnom Penh, Cambodia; 6 Epidemiology and Infectious Diseases Unit, Institut Pasteur de Dakar, Dakar, Senegal; 7 Paris Descartes University and AP-HP, Necker-Enfants Malades University Hospital, Department of Neonatology, Paris, France; Institut National de la Recherche Agronomique, FRANCE

## Abstract

In low and middle income countries (LMICs), where the burden of neonatal sepsis is the highest, the spread of extended spectrum beta-lactamase-producing enterobacteriaceae (ESBL-PE) in the community, potentially contributing to the neonatal mortality, is a public health concern. Data regarding the acquisition of ESBL-PE during the neonatal period are scarce. The routes of transmission are not well defined and particularly the possible key role played by pregnant women. This study aimed to understand the neonatal acquisition of ESBL-PE in the community in Madagascar. The study was conducted in urban and semi-rural areas. Newborns were included at birth and followed-up during their first month of life. Maternal stool samples at delivery and six stool samples in each infant were collected to screen for ESBL-PE. A Cox proportional hazards model was performed to identify factors associated with the first ESBL-PE acquisition. The incidence rate of ESBL-PE acquisition was 10.4 cases/1000 newborn-days [95% CI: 8.0–13.4 cases per 1000 newborn-days]. Of the 83 ESBL-PE isolates identified, *Escherichia coli* was the most frequent species (n = 28, 34.1%), followed by *Klebsiella pneumoniae* (n = 20, 24.4%). Cox multivariate analysis showed that independent risk factors for ESBL-PE acquisition were low birth weight (adjusted Hazard-ratio (aHR) = 2.7, 95% CI [1.2; 5.9]), cesarean-section, (aHR = 3.4, 95% CI [1.7; 7.1]) and maternal use of antibiotics at delivery (aHR = 2.2, 95% CI [1.1; 4.5]). Our results confirm that mothers play a significant role in the neonatal acquisition of ESBL-PE. In LMICs, public health interventions during pregnancy should be reinforced to avoid unnecessary caesarean section, unnecessary antibiotic use at delivery and low birth weight newborns.

## Introduction

Since 2000, the number of neonatal deaths has decreased from 3.9 million to 2.7 million [1]. Nevertheless, this decline has been slower than the post-neonatal under-five mortality [2] and significant improvements in neonatal health still have to be achieved. Bacterial infections are a leading cause of neonatal deaths in countries with very high under five mortality rate [3,4]. In 2014, it has been estimated that 6.9 million cases of neonatal possible severe bacterial infection (pSBI) occurred in low or middle-income countries (LMICs) [5].

In LMICs, neonatal infections are mainly caused by Enterobacteriaceae, more specifically *Escherichia coli* and *Klebsiella pneumoniae* [6,7]. One important driver of unfavorable outcome in infections caused by these bacteria is multidrug resistance which challenges appropriate therapy [8]. Of particular concern, the extended-spectrum beta-lactamase-producing enterobacteriaceae (ESBL-PE), are the most frequently isolated multidrug resistant bacteria [6,9–11]. First reported in hospital settings, ESBL-PE emerged in the community since the early 2000s [12]. ESBL-PE are resistant to beta-lactams and cephalosporins [13]. Carbapenems remain the last resort to treat pediatric ESBL-PE infections which is onerous and often unavailable in LMICs [14–16].

Enterobacteriaceae are known to colonize the digestive tract [17]. After birth, the intestinal tract of newborns is colonized by the maternal and environment flora [18] which represents the first step for potential neonatal infections [19]. However, data regarding the acquisition of ESBL-PE during the neonatal period are scarce [20]. Bacteria that first colonize the neonatal gut are from different sources (maternal, health-care facilities, community) and the routes of transmission are not well defined [21].The maternal carriage of ESBL-PE is likely to play a significant role in the colonization and/or infection of newborns in the first week of life [22].

In Madagascar, a recent study among pregnant women at delivery in the community showed a significant prevalence (18.5%) of ESBL-PE carriage, and one strain of *Klebsiella pneumoniae* isolated was a New Delhi metallo-beta-lactamase-1 producer, indicating that pregnant women may represent a substantial source for transmission of multiresistant *Enterobacteriaceae* to neonates [23].

The aim of this study was to understand the neonatal acquisition of ESBL-PE colonization in the community in Madagascar.

## Methods

The study was reviewed and approved by the Ethics Committee of the Malagasy Public Health Ministry (no. 113-MSANP/CE 03 November 2014) before the study began and was authorized by the Institut Pasteur in Paris. Written informed consents were obtained from all participants.

### Study design

**Fig 1:**
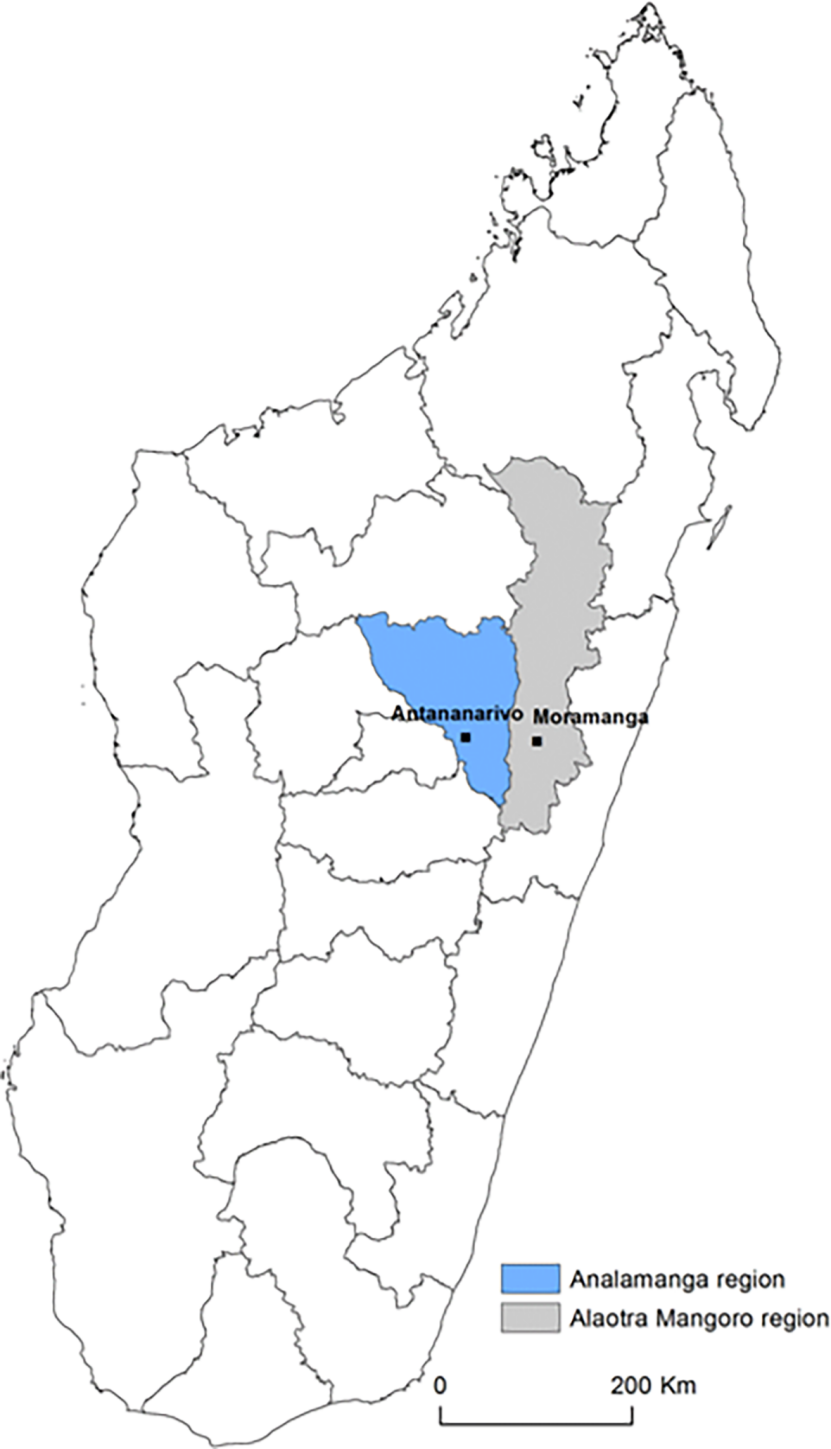
Study areas.

This study was nested within the BIRDY program (“Bacterial Infections and antibiotic Resistant Diseases among Young children in low income countries”, http://www.birdyprogram.org). The study design of the BIRDY program has already detailed elsewhere [24]. Briefly, the present study took place between October 1^st^, 2015 and September 30^th^, 2016 in two distinct areas in Madagascar: an urban area, Antananarivo, and a semi-rural area, Moramanga (110 km east from Antananarivo) (Fig 1). All pregnant women of the study areas were identified and were enrolled in the study during their third trimester of pregnancy. An active monitoring of the pregnant women was conducted to ensure the enrollment of neonates at birth.

The mother’s sociodemographic, medical and obstetric characteristics and delivery information were also collected as well as the newborn’s anthropometric measurements and APGAR score.

Newborns were included at birth and systematic home visits were planned twice in the first week of life; the first visit occurring within three days after delivery and then weekly in the first month (day 0, day 3, day 7, day 14, day 21 and day 28). At each visit, stools sample or endorectal swabs were collected. We recorded using standardized questionnaires orally administered to the mothers factors potentially associated with the risk of ESBL-PE acquisition, including habitat type, number of rooms, number of household members, parity, diet and hygiene habits, contact with animals, animal ownership, animal husbandry, hospitalization and consumption of antibiotics in the previous days, latrines [12,25–28].

Maternal fresh stools were collected at delivery or shortly after delivery in case of home delivery to detect ESBL-PE. Six stools samples or endorectal swabs from the newborns were obtained during the first month of life starting with the meconium when available, then 2 others during the first week and weekly for the 3 remaining weeks of the first month of life. In case of hospitalization of the newborn, a sample was taken at the entrance and every 7 days during hospitalization. Another sample was taken before discharge from the hospital if hospitalization was less than 7 days.

### Microbiological analysis

All samples were analyzed in the laboratory of Experimental Bacteriology unit at the Institut Pasteur in Madagascar. If stools were collected, 1g of sample was suspended in 9ml of physiologic water and dilution in 1/10 was prepared. If endorectal swabs were collected, the swab was suspended in 1ml of physiologic water then vortexed.

Samples were cultivated on CHROMagar ESBL (CHROMagar, Paris, France). The presence of ESBLs in isolates was confirmed by the double-disk synergy test (DDST) [29]. DDST was performed by placing the disk of cefotaxime (30 μg), ceftazidime (30 μg), and combination of amoxicillin/clavulanic acid (20 μg/10 μg) on a lawn culture of bacteria on Muller-Hinton agar plate, with a 20 mm distance between each disk from center to center. Then, plates were incubated at 37°C for 18–24 hrs. Enhancement of the inhibition zone between the disks containing clavulanic acid and cefotaxime or ceftazidime indicated the presence of ESBL production. E. coli ATCC 25922 and K. pneumoniae ATCC 700603 were used as internal quality control strains. Each colony morphotype was identified by mass spectrometry MALDI-TOF, Bruker. Antimicrobial susceptibility testing was performed on each isolate. Amoxicillin, ticarcillin, ticarcillin-clavulanic acid, cefalotin, amoxicillin-clavulanic acid, aztreonam, cefotaxim, ceftazidime, cefepime, imipenem, ertapenem, cefoxitin, cefuroxim, gentamicin, nalidixic acid, ciprofloxacin were tested. The phenotype interpretation was done according to the Antibiogram Committee of the French Society of Microbiology (CA-SFM) guidelines 2015[29].

### Statistical analysis

Stata version no.14 (Stata Corp., College Station, TX) was used for all statistical analyses. Differences in proportions and means were compared using the chi2 and the student test, respectively. A *P* -*value* of < 0.05 was considered statistically significant.

We made the assumption that at birth the newborn gut was not colonized by ESBL-PE [18,30]. Also, newborns that had their first sample after the 2 first weeks of life were not included in the survival analysis [31]. We defined low birth weight (LBW) as a birth weight under 2500 g.

Our primary outcome was defined as the first acquisition of ESBL-PE occurring during the first month of life of the newborn; follow-up was thus censored after the first month, or at the date of death, date of withdrawal, or date of last visit for the newborns lost to follow-up, whichever occurred first. We calculated incidence rates of the first acquisition of ESBL-PE per 1000 newborn-days. The ESBL-PE acquisition curves were obtained by the Kaplan–Meier method and compared using the log-rank statistic. To identify factors associated with the first ESBL-PE acquisition, we used a Cox proportional hazard model. We considered variables potentially associated with ESBL-PE acquisition, such as maternal ESBL-PE colonization, antibiotic consumption, and hygiene.

For all factors included in the Cox model, the proportional hazard assumption was validated using a test on Schoenfeld residuals. All factors associated with the outcome with a *P*-*value* <0.20 in univariate analysis were entered in the multivariate model. A backward selection procedure was applied to identify factors independently and significantly associated with the outcome. We also tested an interaction between maternal ESBL-PE colonization and cesarean delivery; and antibiotic per-partum, respectively. A *P*-value <0.05 was considered significant.

## Results

### Characteristics of the study population

**Fig 2:**
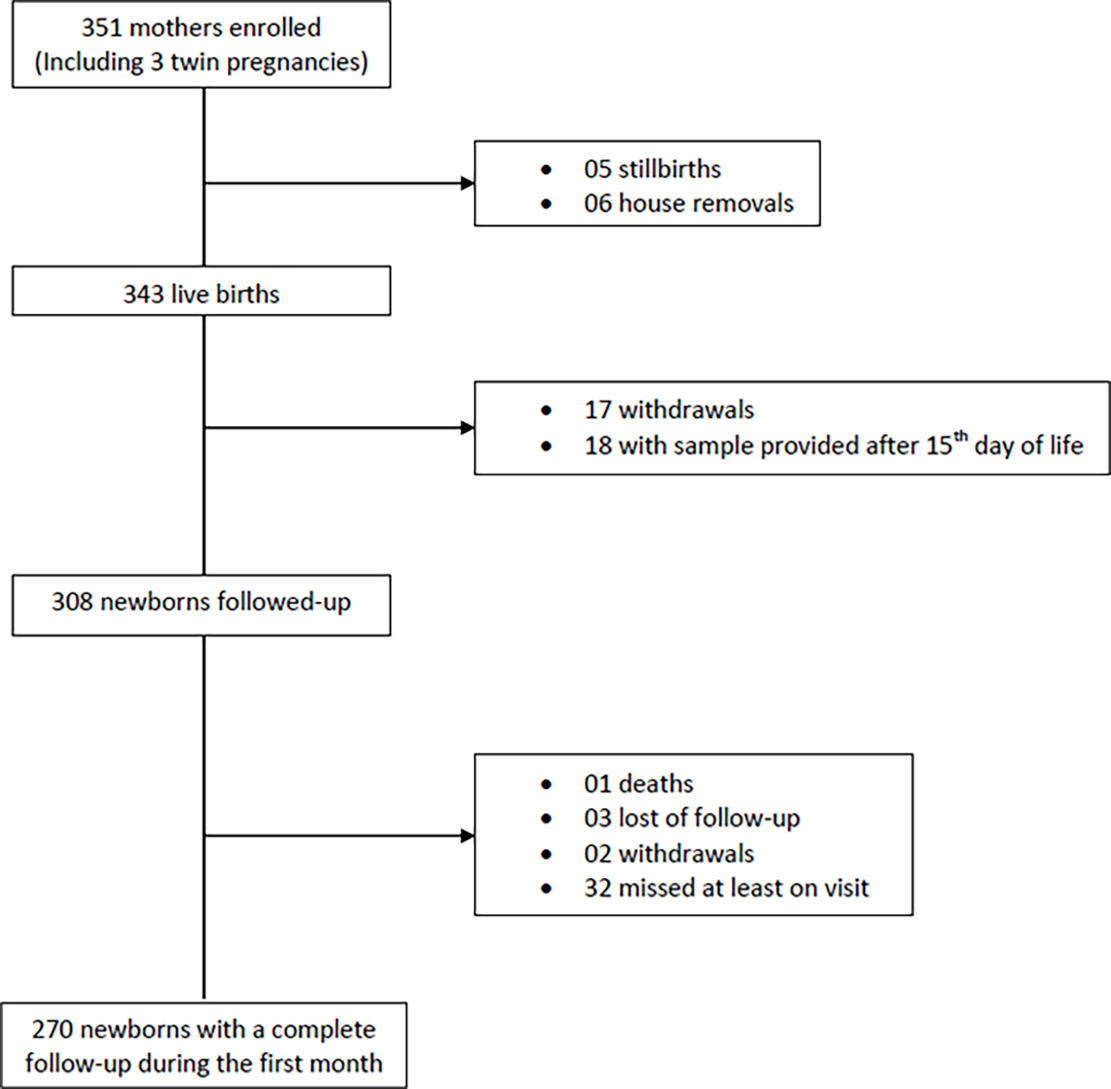
Flow-chart of the study participants.

Of the 351 pregnant women included in the study, 340 gave birth to 343 live newborns who were included in the study (Fig 2). At the end of the study, the mean number of visits was 4.96 (standard-deviation (SD) = 0.04) and 270 newborns had a complete follow-up.

Among the 1755 samples collected in neonates, 678 (39%) were stool samples and 1077 (61%) were endorectal swabs. And among the 275 maternal samples, 177 (64%) were stool samples and 98 (36%) were endorectal swabs.

Table 1 presents the general characteristics of the mothers and newborns. On average, mothers were 27.5 years of age (range 26.8–28.2), 29.1% were primigravidae. More than one-half (54.7%) attended to partial secondary school and more than two third (67.4%) were unemployed. More than 90% used outside toilets and more than one quarter (25.6%) did not have access to electricity. A total of 153 (44.6%) pregnant women gave birth at home. Of the 343 newborns included in the study, 48.1% were male. Their mean birth weight was 3067.6 g (SD = 23.8, range: [1610–4200]); and 7.3% of them had a low birth weight. Among the 308 newborns followed-up, eleven (3.6%) were hospitalized and 5 (1.6%) took antibiotics during their first month of life. Of the 275 mothers with collected stool samples, 54 (19.6%) were colonized with ESBL-PE. The majority were *Escherichia coli* (n = 28, 49.1%) and *Klebsiella pneumoniae* (n = 6, 10.5%) S1 Table.

**Table 1:** Characteristics of the mothers and newborns.

	Total	Urban area	Semi-urban area	P^a^
***Mothers***	***340 (100)***	***103 (30*.*3)***	***237 (69*.*7)***	
**Age**	n (%)	n (%)	n (%)	
Mean (SD^b^)	27.5 (6.5)	26.7 (5.8)	27.9 (6.7)	0.5
Median (IQR^c^)	26 (23; 32)	25 (23; 29)	26 (23; 32)	
**Marital status**				
Single or divorced	18 (5.3)	2 (1.9)	16 (6.8)	0.1
Married or consensual union	322 (94.7)	101 (98.1)	222 (93.3)	
**ESBL colonisation**	275 (100)	77 (28)	198 (72)	0.7
Yes	54 (19.6)	16 (20.8)	38 (19.2)	
No	221 (80.4)	61 (79.2)	160 (80.8)	
**Education**				
No education or primary	77 (22.7)	18 (17.5)	59 (24.9)	0.1
Partial secondary	186 (54.7)	56 (54.4)	130 (54.9)	
Complete secondary or University	77 (22.6)	29 (28.1)	48 (20.2)	
**Parity**				
Primigravidae	99 (29.1)	33 (32.0)	66 (27.9)	0.4
Multigravidae	241 (70.9)	70 (68.0)	171 (72.1)	
**Toilets facilities**				
Inside access	24 (7.1)	13 (12.6)	11 (4.6)	**0.008**
Outside access	316 (92.9)	90 (87.4)	226 (95.4)	
**Electricity access**				
Yes	253 (74.4)	89 (86.4)	164 (69.2)	**0.001**
No	87 (25.6)	14 (13.6)	73 (30.8)	
**Profession**				
Unemployed	229 (67.4)	63 (61.2)	166 (70.0)	0.06
Manual employment	101 (29.7)	34 (33.0)	67 (28.3)	
Office jobs	10 (2.9)	6 (5.8)	4 (1.7)	
**Person who followed pregnancy**				
No follow-up	10 (2.9)	2 (1.9)	8 (3.4)	0.9
Traditional birth attendant	7 (2.1)	2 (1.9)	5 (2.1)	
Health care worker^d^	323 (95.0)	99 (96.2)	224 (94.5)	
***Newborns***	***343 (100)***	***105 (30*.*6)***	***238 (69*.*4)***	
**Sex**				
Male	165 (48.1)	52 (49.5)	113 (47.5)	0.7
**Place of delivery**				
Health care center	190 (55.4)	74 (70.5)	116 (48.7)	**>0.001**
Home	153 (44.6)	31 (29.5)	122 (51.3)	
**Cesarean delivery**				
Yes	29 (8.5)	17 (16.2)	12 (5.0)	**0.001**
No	314 (91.5)	88 (83.8)	226 (95.0)	
**Difficult birth**				
Yes	25 (7.3)	13 (12.4)	12 (5.0)	**0.016**
No	318 (92.7)	92 (87.6)	226 (95.0)	
**Weight at delivery (gr)**	329 (100)	101 (30.7)	228 (69.3)	
< 2500	24 (7.3)	15 (14.9)	9 (3.9)	**>0.001**
> = 2500	305 (92.7)	86 (85.1)	219 (96.1)	
**Hospitalization during the first month of life**	308 (100)	89 (28.9)	219 (71.1)	
No	297 (96.4)	82 (92.1)	215 (98.2)	**0.01**
Yes	11 (3.6)	7 (7.9)	4 (1.8)	
**Antibiotics intake during the first month of life**	308 (100)	89 (28.9)	219 (71.1)	
No	303 (98.4)	88 (98.9)	215 (98.2)	1
Yes	5 (1.6)	1 (1.1)	4 (1.8)	

^a^p, p-value

^b^SD, Standard deviation

^c^IQR, Interquartile range.

^d^Health care worker: a doctor, a midwife or a nurse.

### ESBL-PE acquisition in newborns

A total of 83 ESBL-PE isolates were identified during the follow-up. *Escherichia coli* was the most frequent species (n = 28, 34.1%), followed by *Klebsiella pneumoniae* (n = 20, 24.4%). Eleven strains remained unidentified. When we considered the first acquisition only (n = 55), *Escherichia coli* and *Klebsiella pneumoniae* remained the most prevalent pathogens (n = 17, 30.9% and n = 14, 25.5%, respectively) (see S2 and S3 Tables). Overall, thirty five newborns were excluded from the survival analysis (18 with sample provided after 15^th^ day of life, 17 withdrawls (Fig 2). There was no statistical difference between the characteristics of these newborns and those included in the survival analysis.

**Fig 3:**
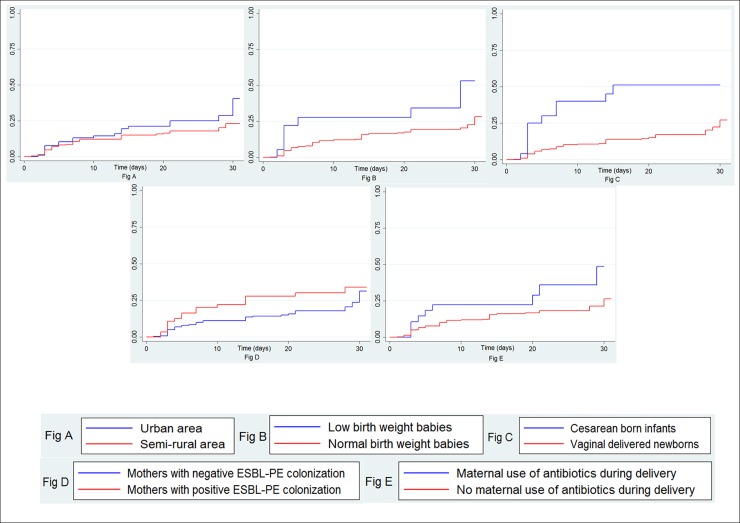
**Cumulative incidences**according to study areas (Fig 3A), newborn’s weight at birth (Fig 3B), mode of delivery (Fig 3), maternal ESBL-PE colonization status (Fig 3D) and antibiotics use during delivery (Fig 3E).

The overall incidence of ESBL-PE first acquisition was 10.4 per 1000 newborn-days [95% confident interval (CI) = 8.0; 13.4]. The incidences were 13.7 [8.8; 21.3] and 9, 2 [6.7; 12.7] in the urban and semi-rural areas, respectively. We did not find any difference in the incidence of acquisition between the urban and semi-rural areas (log rank test, p = 0.22). ESBL-PE acquisition curves according to study areas, newborn’s weight, mode of delivery, maternal ESBL-PE colonization status and antibiotics use during delivery are shown in Fig 3A, Fig 3B, Fig 3C, Fig 3D and Fig 3E, respectively. In univariate Cox analysis (Table 2), cesarean-born babies (Hazard-ratio (HR) = 3.5, 95% CI [1.7; 6.8]) or newborn from a mother who received an antibiotic during delivery (HR = 2.1, 95% CI [1.0; 4.2]) were at higher risk of ESBL-PE acquisition than vaginally born babies or newborn from a mother who did not receive an antibiotic during delivery, respectively. Compared to babies born from a mother not colonized with ESBL-PE, children born from an ESBL-PE colonized mother had an increased risk of acquiring ESBL-PE (HR = 1.7, 95% CI [1.0; 3.0]). Low birth weight was significantly associated with a higher risk of ESBL-PE acquisition (HR = 2.6, 95% CI [1.2; 5.5]) compared to normal weight babies at birth.

**Table 2:** Cox proportional hazard analysis of ESBL-PE acquisition.

Variable		Univariate analysis		Multivariate analysis	
	Person-days	Crude HR^a^ [95% CI^b^]	*P*^c^	Adjusted HR [95% CI]	*p*
**Low birth weight**			***0*.*01***		***0*.*01***
No (> = 2500gr)	5300	Ref.^d^		Ref.	
Yes (<2500gr)	324	2.6 [1.2; 5.5]		2.7 [1.2 ; 5.9]	
**Maternal job**			*0*.*21*		
No Job	3900	Ref.			
Manual job	1500	1.0 [0.6; 1.9]			
Office job	199	2.5 [1.0; 6.5]			
**Latrine type**			*0*.*41*		
Outside without flush	5100	Ref.			
Outside with flush	137	0.7 [0.1; 5.4]			
Inside without flush	176	0.5 [0.1; 3.8]			
Inside with flush	232	2.1 [0.9; 5.3]			
**Habitat type**			*0*.*46*		
Room shared with other household	4389	Ref.			
House shared with other household	500	0.8 [0.4; 1.4]			
Individual house	410	1.3 [0.2; 2.5]			
**Number of rooms**			*0*.*63*		
1 room		Ref.			
2 rooms and more		1.1 [0.6; 1.9]			
**Number of household members**			*0*.*16*		
Less than 5 members	4500	Ref.			
5 members and more	1097	1.01 [0.5; 1.7]			
**Animal ownership**			0.32		
No	5370	Ref.			
Yes	214	1.7 |0.2; 10.6]			
**Site**			*0*.*19*		0.96
Semi-rural site	4100	Ref.		Ref.	
Urban site	1500	1.4 [0.8; 2.5]		1.0 [0.5; 1.8]	
**Mode of delivery**			***<0*.*001***		***0*.*001***
Vaginal delivery	5300	Ref.		Ref.	
Cesarean	320	3.5 [1.8; 6.8]		3.4 [1.7 ; 7.1]	
**Supervising person at delivery**			*0*.*14*		
Non medical staff	1700	Ref.			
Medical staff	3800	1.6 [0.8 ; 2.9]			
**Maternal ESBL-carriage**			0.06		*0*.*09*
No	4000	Ref.		Ref.	
Yes	1100	1.7 [1.0 ; 3.0]		1.6 [0.9 ; 2.9]	
**Antibiotic during delivery**			***0*.*04***		***0*.*04***
No	5200	Ref.		Ref.	
Yes	423	2.1 [1.0; 4.2]		2.2 [1.1; 4.5]	
**Place of delivery**			*0*.*09*		
Home	2600	Ref.			
Health-care facilities	3000	1.6 [0.9 ; 2.7]			
**Hospitalization during the first month of life**			0.23		
No	5500	Ref.			
Yes	84	2.7 |0.62; 11.2]			
**Antibiotics intake during the first month of life**			0.54		
No	5500	Ref.			
Yes	58	2.0 [0.27;14.7]			

^a^HR, Hazard Ratio

^b^95% CI, 95% Confidence Interval

^c^p, p-value

^d^Ref., reference.

In multivariate analysis (Table 2), factors independently associated with a higher risk of first ESPL-PE acquisition included low birth weight (adjusted Hazard-ration (aHR) = 2.7, 95% CI [1.2; 5.9]), cesarean delivery (aHR = 3.4, 95% CI [1.7; 7.1]) and maternal use of antibiotics at delivery (aHR = 2.2, 95% CI [1.1; 4.5]). Maternal ESBL-PE colonization status was no longer statistically significant after adjustment. We found no statistically significant interactions.

## Discussion

This study showed an overall incidence of 10.4 ESBL-PE acquisitions per 1000 newborn-days within a cohort of newborns in the community in Madagascar. Also, we found that low birth weight, cesarean delivery and maternal use of antibiotic at delivery were major risk factors of acquiring ESBL-PE during the first month of life.

To our best knowledge, this is the first community-based estimate of the incidence of ESBL-PE acquisition in newborns in a LMIC. In these settings, there are very few data from the community and the majority of published studies estimated a prevalence (ranging from 10.0% to 46.0%) which is less accurate than an incidence [12,23,26,32–36]. In addition, most studies [37–40] on the acquisition of ESBL-PE were conducted in neonatal intensive care units where ESBL-PE are more likely to be hospital-acquired and where neonates have serious illness, are more exposed to broad-spectrum antibiotics and are thus not representative of the general population. In these studies, two swabs were usually performed: the first one at admission and the second one at discharge which does not allow an accurate incidence estimation. Our estimate is just below the lower limit of monthly incidence of ESPL-PE acquisition [12 and 53 cases per 1000 patient-days] estimated by Mammina et al. in a neonatal intensive care unit in Italy [31]. Thus, this finding highlights that, even in neonates not particularly exposed to hospital environment, ESBL-PE spread is significant and very fast from the very beginning of life.

We found that cesarean delivery is a risk factor of ESBL-PE acquisition which is concordant with others studies on ESBL-PE infection or carriage [32,41]. Babies delivered by cesarean section are more at risk of handling by health personnel and have generally longer stay in hospital than vaginally delivered newborns. These two factors might increase the risk of acquiring ESBL-PE. Moreover, birth by cesarean section deprives newborns from maternal vaginal and gut flora exposure and may influence the newborn microbiome development [42,43]. Recent studies show that the microbiota play a key role in the protection against infectious diseases [44]. One plausible explanation to our finding is that the maternal microbiota might also play a role in the ESBL-PE acquisition during the first month of life. Concordantly, it has been shown that infants delivered by cesarean section have longer ESBL-PE carriage compared to those vaginally delivered [45].

The rate of cesarean section in low-income countries remains low compared to those observed in high- and middle-income countries [46–49]. However, in LMICs also, cesarean section might be performed while it is not medically indicated, with negative consequences [50]. Our result provides an additional argument to avoid unnecessary cesarean sections.

In the present study, pregnant women were included in their third trimester of pregnancy, which rendered difficult an accurate measurement of gestational age, as none of the included women had a first-trimester ultrasound. Therefore, in our analysis, we used LBW instead, which can be either the consequence of prematurity or intra-uterine growth retardation, or both. We showed that low birth weight babies were at increased risk of acquiring ESBL-PE whatever the ESBL-PE status of the mother. Among the 18 LBW babies with collected stool samples, seven were colonized by ESBL-PE, and only one had been hospitalized among the seven colonized babies, suggesting that the colonization was likely not hospital-acquired. Prematurity has been shown to be a risk factor for acquisition of ESBL-PE [51], whereas to our knowledge the role of intra-uterine growth retardation has never been studied. More studies are then needed to understand which is the underlying pathway of ESBL-PE acquisition in LBW babies, who are consequently more at risk to develop a potentially fatal drug-resistant infection.

Antibiotics during delivery are administered for various reasons [52], including for preventing the transmission of maternal group B *streptococcus* (GBS) to newborns. However, in our study, all mothers (n = 28) who received antibiotics during delivery had fetid amniotic fluid and/or premature rupture of the membrane and/or fever during delivery. Although these factors did not indicate that the mother was definitively infected, the delivery of these women might occurred in a septic context, which might increases the risk of an earlier ESBL-PE acquisition in newborns.

It is the first time, to our knowledge, that maternal use of antibiotics at delivery has been shown as a risk factor of ESBL-PE acquisition in newborns. Penicillin A, the most common antibiotics used [53], is able to cross the placenta and thus to be transmitted to the fetus. Consequently, it is likely that the newborn also received a part of maternal antibiotic and may explain why infants born from mothers who had antibiotics during delivery might be at increased risk of an earlier ESBL-PE acquisition in newborns.

It is well known that unnecessary use of antibiotics has subsequent impact on drug resistance and cost of healthcare in both LMICs and high-resource countries [54–58]. Measures to prevent it are currently under development (rapid diagnostic test) or have already been implemented, for example: through integrated community case management [59]. Thus, our findings support that reinforcing antibiotic stewardship is of the utmost importance. We did not find that hospitalization or antibiotic intake was associated with an increased risk of the first acquisition of ESBL-PE in newborns. As our study was community-based, few newborns were hospitalized (3.6%) or took antibiotics during their first month of life (1.6%) and therefore less exposed compared to neonates of the studies which are for the majority hospital-based [51,60–63]. In community-settings, risk factors might therefore be different and the mother and more specifically pregnancy conditions are predominant for ESBL-PE acquisition in neonates.

Although marginally significant in the univariate analysis, we did not found that maternal ESBL-PE colonization significantly increases the risk of ESBL-PE carriage for the newborns in the multivariate model. When we considered the acquisition of ESBL-PE during the first week of life, only 3 pairs of mother/infant carried the same pathogen. Although other studies found that maternal ESBL-PE was associated with ESBL-PE colonization in newborns [64,65], this finding suggests that mother to child transmission during delivery might not play such a significant role in the acquisition of colonization in the first week of life.

However, we cannot exclude that the none statistically significant associations between hospitalization, use of antibiotics during the first month of life, maternal ESBL-PE carriage and ESBL-PE acquisition in neonates, may be due to a lack of statistical power. Therefore, the interpretation of these results must be cautious.

Our study had some limitations. Six anal swabs were planned during the follow-up. For ethical reasons, it was not possible to perform more frequent swabs. Therefore, the time of acquisition of ESBL-PE might have occurred earlier than at the time of the positive ESBL-PE swab, within the span-time of two consecutive swabs. However, this inaccurate estimation of the acquisition date concerns all included newborns in the same way and is not likely to have had an impact on the risk factors we found associated with a higher risk of ESBL-acquisition. Also, the exposures to LBW, cesarean section and maternal carriage do not change over time and occur before and at the time of birth.

A great majority of the newborns (75.6%) had their first swab within the 3 first days. However, due to logistical issues, 75 newborns could not have their sample taken during this period. Among them, seven had only one sample during their whole follow-up (missed visits or lost-to follow-up). In the 68 remaining newborns, all subsequent samples were negative, suggesting that the missing sample within the three first days of life was likely negative. We are then confident that these limitations may not impact the associations we found.

Eleven Gram negative bacteria (GNB) in newborns stool samples and 14 GNB in mothers stool samples could not be identified by the MALDI-TOF SM. They were all Gram-negative, oxidase-negative, aerobic rods. The scores provided by the MALDI-TOF mass spectrometer were too low (1.2–1.7) and did not allow us to identify the species but gave us an idea about the bacteria genus (Enterobacteriaceae: *Acinetobacter sp*., *Enterobacter sp*., *Kluyvera sp*., *Raoultella sp*. and other Gram negative rods such as *Stenotrophomonas sp*.).

We did not have collect data on ESBL-PE colonization in household members. However, characteristics reflecting a potential transmission from the household members (habitat type, number of room, number of household members) were not significantly associated with ESBL-PE acquisition in newborns.

## Conclusions

To our knowledge, we provide the first community-based estimate of the incidence of ESBL-PE acquisition in newborn in LMICs. This incidence is substantial; one other striking result of our study is the key role played by the mother in the acquisition of ESBL-PE by the neonates. We highlight that the course of pregnancy, including delivery and the previous months before birth, may influence the ESBL-PE acquisition and consequently potential subsequent neonatal infection. Our results reinforce that enhancing public-health interventions to promote antibiotic stewardship and to better monitor pregnancy to avoid unnecessary caesarean section, unnecessary antibiotic use at delivery and low birth weight newborns is of utmost importance.

## Supporting information

S1 File BIRDY study group. (DOCX)

S1 Table Pathogens isolated from maternal stool samples. (PDF)

S2 Table Pathogens isolated from neonatal stool samples during the follow-up. (PDF)

S3 Table Pathogens isolated from neonatal stool sample at the first acquisition. (PDF)

S4 Table Antibiotic susceptibility in ESBL strains isolated from neonates. (PDF)

S5 Table Antibiotic susceptibility in ESBL strains isolated from mothers. (PDF)
