# Supplementary material for: Acquisition of extended spectrum beta-lactamase-producing enterobacteriaceae in neonates: A community based cohort in Madagascar
Source: PLoS One. 2018 Mar 1;13(3):e0193325. doi: 10.1371/journal.pone.0193325 (PMC5832238; doi:10.1371/journal.pone.0193325)
Supplement: S1 File — (DOCX) [file pone.0193325.s001.docx]

**BIRDY study group**

**Madagascar**

Bodonirina Tanjona RAHELIARIVAO, Epidemiology & Public Health Unit**,** Institut Pasteur Madagascar

Frédérique RANDRIANIRINA, Centre de Biologie Clinique, Institut Pasteur Madagascar

**Cambodia**

Laurence Borand, Epidemiology & Public Health Unit, Institut Pasteur du Cambodge, Phnom Penh, Cambodia

Alexandra Kerléguer, Medical Biology Unit, Institut Pasteur du Cambodge, Phnom Penh, Cambodia

Thida Chon, Epidemiology & Public Health Unit, Institut Pasteur du Cambodge, Phnom Penh, Cambodia

Sok Touch, Director of CDC Department (ret.) at Ministry of Health Cambodia

Arnaud Tarantola, Epidemiology & Public Health Unit, Institut Pasteur du Cambodge, Phnom Penh, Cambodia

Sophie Goyet, Epidemiology & Public Health Unit, Institut Pasteur du Cambodge, Phnom Penh, Cambodia

Siyin Lach, Epidemiology & Public Health Unit, Institut Pasteur du Cambodge, Phnom Penh, Cambodia

Veronique Ngo, Epidemiology & Public Health Unit, Institut Pasteur du Cambodge, Phnom Penh, Cambodia

**Senegal**

Muriel Vray, Epidemiology & Public Health Unit, Institut Pasteur de Dakar, Senegal

Marguerite Diatta, Epidemiology & Public Health Unit, Institut Pasteur de Dakar, Senegal

Joseph Faye, Epidemiology & Public Health Unit, Institut Pasteur de Dakar, Senegal

Abibatou Ndiaye, Institut Pasteur de Dakar, Senegal

Vincent Richard, Epidemiology & Public Health Unit, Institut Pasteur de Dakar, Senegal

Abdoulaye Seck, laboratory of medical biology, Institut Pasteur de Dakar, Senegal

Raymond Bercion, laboratory of medical biology , Institut Pasteur de Dakar

Amy GASSAMA SOW, Unit of Experimental Bacteriology, Institut Pasteur de Dakar

Jean Baptiste DIOUF, Hôpital Roi Baudoin

Pape Samba DIEYE, District Sanitaire de Guédiawaye

Balla SY, District Sanitaire de Sokone

Bouya NDAO, District Sanitaire de Sokone

**France**

Maud Seguy, Department of International Affairs, Institut Pasteur Paris

Laurence Watier, Inserm, UVSQ, Institut Pasteur_ UMR 1181 « Biostatistics, Biomathematics, Pharmacoepidemiology and Infectious Diseases » (B2PHI), Paris, France

Abdou Armya Youssouf, Inserm, UVSQ, Institut Pasteur_ UMR 1181 « Biostatistics, Biomathematics, Pharmacoepidemiology and Infectious Diseases » (B2PHI), Paris, France

Nadimpalli Maya Inserm, UVSQ, Institut Pasteur_ UMR 1181 « Biostatistics, Biomathematics, Pharmacoepidemiology and Infectious Diseases » (B2PHI), Paris, France
