# Supplementary material for: Acquisition of extended spectrum beta-lactamase-producing enterobacteriaceae in neonates: A community based cohort in Madagascar
Source: PLoS One. 2018 Mar 1;13(3):e0193325. doi: 10.1371/journal.pone.0193325 (PMC5832238; doi:10.1371/journal.pone.0193325)
Supplement: S1 Table — (PDF) [file pone.0193325.s002.pdf]

PATHOGENS<br>n= 57	Frequency	%
Escherichia coli	28	49.12
Klebsiella pneumoniae	6	10.53
Enterobacter cloacae	3	5.26
Acinetobacter baumannii	3	5.26
Acinetobacter nosocomialis	1	1.75
Acinetobacter guillouiae	1	1.75
Acinetobacter calcoaceticus	1	1.75
Unidentified	14	24.56
