# Supplementary material for: Acquisition of extended spectrum beta-lactamase-producing enterobacteriaceae in neonates: A community based cohort in Madagascar
Source: PLoS One. 2018 Mar 1;13(3):e0193325. doi: 10.1371/journal.pone.0193325 (PMC5832238; doi:10.1371/journal.pone.0193325)
Supplement: S2 Table — (PDF) [file pone.0193325.s003.pdf]

PATHOGENS<br>n= 83	Frequency	%
Escherichia coli	28	34.15
Klebsiella pneumoniae	20	24.39
Enterobacter cloacae	5	6.10
Acinetobacter baumannii	7	8.54
Acinetobacter nosocomialis	4	4.88
Acinetobacter guillouiae	1	1.22
Acinetobacter calcoaceticus	1	1.22
Acinetobacter pitii	1	1.22
Citrobacter freundii	1	1.22
Klebsiella oxytoca	1	1.22
Pichia norvegensis	1	1.22
Kluyvera ascorbata	1	1.22
Unidentified	11	13.41
