# Supplementary material for: Acquisition of extended spectrum beta-lactamase-producing enterobacteriaceae in neonates: A community based cohort in Madagascar
Source: PLoS One. 2018 Mar 1;13(3):e0193325. doi: 10.1371/journal.pone.0193325 (PMC5832238; doi:10.1371/journal.pone.0193325)
Supplement: S3 Table — (PDF) [file pone.0193325.s004.pdf]

**PATHOGENS**<br>n= 55	**Frequency**	**%**
Escherichia coli	17	30.91
Klebsiella pneumoniae	14	25.45
Enterobacter cloacae	2	3.64
Acinetobacter baumannii	6	10.91
Acinetobacter nosocomialis	3	5.45
Acinetobacter guillouiae	1	1.82
Acinetobacter calcoaceticus	1	1.82
Acinetobacter pitii	1	1.82
Citrobacter freundii	1	1.82
Pichia norvegensis	1	1.82
Acinetobacter ursigii	1	1.82
Kluyvera ascorbata	1	1.82
Unidentified	6	10.91
