# Supplementary material for: Acquisition of extended spectrum beta-lactamase-producing enterobacteriaceae in neonates: A community based cohort in Madagascar
Source: PLoS One. 2018 Mar 1;13(3):e0193325. doi: 10.1371/journal.pone.0193325 (PMC5832238; doi:10.1371/journal.pone.0193325)
Supplement: S4 Table — (PDF) [file pone.0193325.s005.pdf]

ESBL Strains	RESISTANCES			
	Distribution	*NAL Ac.	CIPRO	GENTA
n= 83	n(%)	n(%)	n(%)	n(%)
Escherichia coli	28 (33.7)	13 (46.4)	9 (32.1)	10 (35.7)
Klebsiella pneumoniae	20 (24.1)	5 (25.0)	7 (35.0)	12 (60.0)
Enterobacter cloacae	5 (6.0)	2 (40.0)	2 (40.0)	2 (40.0)
Acinetobacter spp.	15 (18.1)	6 (40.0)	7 (46.7)	8 (53.3)
Citrobacter freundii	1 (1.2)	0 (0.0)	0 (0.0)	0 (0.0)
Klebsiella oxytoca	1 (1.2)	0 (0.0)	0 (0.0)	0 (0.0)
Pichia norvegensis	1 (1.2)	0 (0.0)	0 (0.0)	1 (100)
Kluyvera ascorbata	1 (1.2)	0 (0.0)	0 (0.0)	0 (0.0)
Unidentified	11 (13.3)	2 (18.2)	2 (18.2)	5 (45.5)

*NAL Ac.: Nalidixic Acid, **CIPRO**: Ciprofloxacin, **GENTA**: Gentamicin
