# Supplementary material for: Acquisition of extended spectrum beta-lactamase-producing enterobacteriaceae in neonates: A community based cohort in Madagascar
Source: PLoS One. 2018 Mar 1;13(3):e0193325. doi: 10.1371/journal.pone.0193325 (PMC5832238; doi:10.1371/journal.pone.0193325)
Supplement: S5 Table — (PDF) [file pone.0193325.s006.pdf]

PATHOGENS	Distribution	RESISTANCES *Ac. NAL	CIPRO	GENTA
n= 57	n(%)	n(%)	n(%)	n(%)
Escherichia coli	28 (49.1)	12 (42.9)	10 (35.7)	15 (53.6)
Klebsiella pneumoniae	6 (10.5)	2 (33.3)	2 (33.3)	3 (50)
Enterobacter cloacae	3 (5.3)	3 (100)	3 (100)	3 (100)
Acinetobacter spp.	6 (10.5)	2 (33.3)	2 (33.3)	4 (66.7)
Unidentified	14 (24.6)	8 (57.1)	6 (42.9)	6 (42.9)

*__NAL Ac.__ : Nalidixic Acid, **CIPRO**: Ciprofloxacin, **GENTA**: Gentamicin
